# Supplementary material for: An automated pipeline for quantitative T2* fetal body MRI and segmentation at low field
Source: Med Image Comput Comput Assist Interv. Author manuscript; Available in PMC 2024 Sep 20. (PMC7616578; doi:10.1007/978-3-031-43990-2_34)
Supplement: Supplementary Materials [file EMS197977-supplement-Supplementary_Materials.pdf]

# An automated pipeline for quantitative T2* fetal body MRI and segmentation at low field

## 1 Supplementary Information

### 1.1 Multi-Echo Image Acquisition

An example of the differing echo contrasts can be seen in Figure 1.

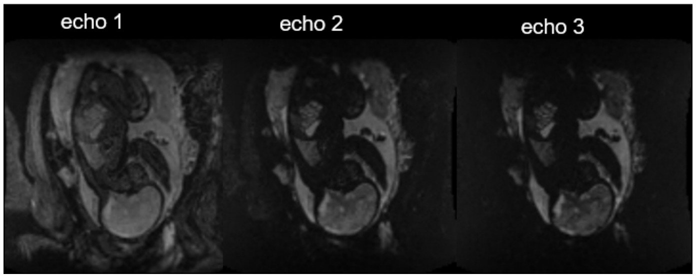


**Fig. 1.** Example echos from the multi-echo sequence. The second echo is used for the 3D volume reconstruction as it has the best contrast in the fetal body organs

### 1.2 Volumetric Growth Curves

Volumetric growth curves (Figure 2) show a strong relationship with gestational age ($R^2 > 0.5$), and a significant increase in volume throughout gestation ($p<0.05$) for all organs.

### 1.3 Demographic Information of Testing Dataset

The pathologies and gestational ages of the segmentation testing dataset can be found in (Table 1)

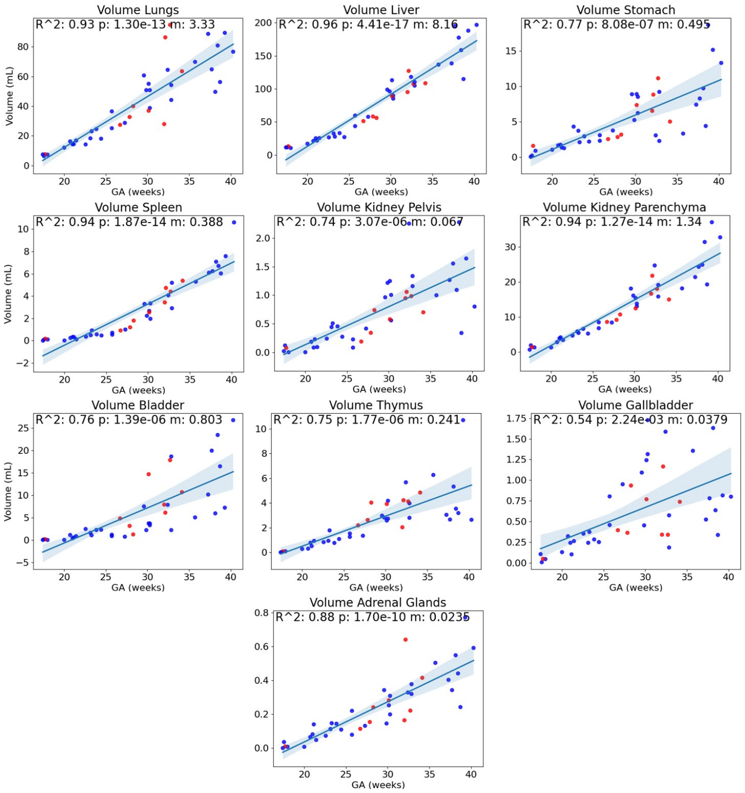


**Fig. 2.** Volumetric growth curves per organ throughout gestation. All organs are found to have a strong ($R^2 > 0.5$) relationship with gestational age

**Table 1.** Pathologies and gestational ages of the cases used in the testing dataset

| Subject | Pathology | Gestational Age (weeks) |
|---|---|---|
| 1 | Control | 35.71 |
| 2 | Control | 20.72 |
| 3 | Pathological | 37.53 |
| 4 | Control | 22.94 |
| 5 | Pathological | 32.14 |
| 6 | Control | 32.43 |
| 7 | Control | 39.23 |
